# Public Health Approach to Problems Related to Excessive and Addictive Use of the Internet and Digital Media

**DOI:** 10.1007/s40429-022-00458-z

**Published:** 2022-12-28

**Authors:** Sulki Chung, Hae Kook Lee

**Affiliations:** 1 Department of Social Welfare, Chung-Ang University, 84 Heuksuk-ro, Dongjak-Gu, Seoul, 06974 Korea; 2 Department of Psychiatry, College of Medicine, Uijeongbu St. Mary’s Hospital, The Catholic University of Korea, 222 Banpo-daero, Seocho-Gu, Seoul, 06591 South Korea

**Keywords:** Public health model, PEAID (problems related to the excessive and addictive use of the Internet and digital media), Information system, Internet use disorder, Problematic Internet use

## Abstract

**Purpose of Review:**

Advances in digital technology and media have provided convenience and advantages in all areas of our daily lives. However, there is a risk of excessive and addictive use, which increases the risk of addiction as a disease and other related mental and physical problems. This article reviews the public health approach to problems related to excessive and addictive use of the Internet and digital media.

**Recent Findings:**

The public health model views addiction as the result of interactions among individuals, digital media, and the environment; therefore, interventions should aim to reduce risk factors and increase protective factors in these three areas. This includes developing and providing evidence-based services according to each individual’s problem type and severity. Regarding interventions for digital media and the environment, restricting accessibility and regulating content may be necessary. This calls for an integrative, comprehensive, and continuous intervention strategy, and to achieve such a framework, we need to establish an information system to monitor the magnitude and patterns of related problems.

**Summary:**

This review suggests a surveillance system that provides a list of evidence-based policies from the public health perspective. Suggestions for an advanced international information, policy, and monitoring system are discussed.

## Introduction

The Internet and digital media have become essential tools for working, learning, and accessing essential public services today. Most of all, digital media connects people in ways never before possible, enabling users to maintain contact across time and distance. However, the Internet and digital media can cause overuse, addiction, and various related physical and mental development problems due to their readily accessible nature [1]. In particular, with the outbreak of the COVID-19 pandemic, studies in Asian countries such as China and India reported an increase in Internet addiction at mild and moderate levels that was related to increased depression and anxiety [2, 3]. Research has been conducted in several countries on the increase in digital media screen time and related mental/behavioral and physical health problems during the COVID-19 pandemic [4, 5]. Increases in screen time, decreases in physical activity, and sleep problems were especially common in children and adolescents [6]. The rise in Internet and digital media use among children and adolescents increased physical health problems (e.g., sleep problems, myopia risk, neck/wrist musculoskeletal problems) and decreased physical activity. Increased risks of mental and behavioral health problems, such as concentration difficulties, depression, anxiety, and attention deficit hyperactivity disorder, have also been reported [7–10]. As with children and adolescents, the World Health Organization (WHO) has cautioned adults to pay attention to decreases in physical activity, sleep cycle changes, and the associated risk of physical problems due to excessive screen time during the COVID-19 pandemic [11].

While the negative aspects of problematic Internet use were particularly visible during the pandemic, they are clearly not limited to this period. These issues related to the use of the Internet should be addressed as a public health concern for several reasons. First, the problems are widely reported in the general population, including in children and adolescents. Second, various types of related harm of varying levels of severity occur depending on the amount and pattern of Internet use. Third, related problems are experienced not only at the individual level but also at the family and community levels [12•]. Based on these insights, on May 25, 2019, the WHO officially included gaming disorder (GD) in the latest (eleventh) revision of the International Classification of Diseases (ICD-11) [13]. This is an official declaration that gaming-related problems are subject to public health intervention. Some experts in the field suggest other conditions related to digital media to be included in the ICD-11 category “other specified disorders due to addictive behaviors” [14]. However, some researchers in media psychology, information technology, and communication science have criticized the inclusion of GD in the ICD-11 [15] despite the results from public health and clinical studies on the harmful effects of the excessive use of games and digital media [16•]. Moreover, with the gaming industry joining the argument against GD being a public health problem, the inclusion of GD remains a social issue to this day [17].

Despite the controversy over the concept of GD as a disease, concerns about the digital media-related health problems that have exploded during the pandemic suggest an urgent need for a public health intervention strategy. Although some suggestions have been made on prevention and intervention strategies for health problems related to the overuse of the Internet and digital media, they remain focused on individuals and have not presented a systematic framework from the public health perspective [18, 19•, 20]. Therefore, this paper intends to present a public health model that explains the addictive use of the Internet and digital media and related problems and to provide an example of an intervention framework and strategies based on this model. In addition, a global monitoring system will be proposed as a tool to disseminate the strategy.

With the recent acceleration of digital technology and infrastructure development, an increasing number of leisure activities depend on online and digital media, and games, gambling, sexual content, and social media communication are converging [21]. Moreover, related problems are experienced at various levels in behavioral, physical, and/or mental health areas beyond the concept of a simple addiction. Therefore, in this paper, we use the term problem related to the excessive and addictive use of the Internet and digital media (PEAID), which combines the existing terms such as game addiction, GD, Internet addiction, and Internet use disorder.

## PEAID as a Problem on a Continuum

Since PEAID is not a scientific term, we borrow the concept of Internet addiction to discuss its spectrum. There is a lack of consensus on the definition of Internet addiction, and the term has been defined differently in various studies. Internet addiction is generally defined as the excessive use of the Internet accompanied by tolerance, withdrawal, and negative consequences [22]. We use PEAID in this paper to represent a pathological state that occurs due to the excessive use of the Internet and digital media. Similar to substance addiction, Internet addiction (and PEAID likewise) should be understood as a problem on a continuum. For example, one of the most frequently used tools to identify the risk of Internet addiction is Young’s Internet Addiction Test. The test categorizes users into four groups: normal users and users with mild, moderate, and severe levels of Internet addiction [23]. In other words, people using the Internet can be grouped into regular users, users at the initial stages of the addiction process, and an addicted group experiencing clear addiction symptoms. Similarly, PEAID is better understood as a problem on a continuous spectrum rather than as a dichotomous disease, and screening and intervention should be performed based on this continuum.

## Public Health Model of PEAID

PEAID is governed by a complex set of interrelating factors and determinants ranging from individual vulnerability and family relationships to addictive features of the Internet and digital media itself and social norms and regulations. To achieve effective prevention and intervention strategies, a model that reflects the complexity of and interactions among the different factors of PEAID is required. Originally designed as a model for infectious disease, the public health model posits that health problems occur as a result of interactions among three main factors [24, 25]. The first is the *agent* that contains certain destructive potential, the second is the *host* who experiences the problem or has individual susceptibility, and the last is the *environmental* context that encourages or discourages the behavior. Hester and Miller [24] explained the public health model as applied to alcohol-related problems, positing that the interaction among alcohol (as the agent with destructive potential), individual differences in susceptibility (the host), and the environment (for example, tolerant culture and availability) needs to be considered in understanding and intervening in alcohol problems. The model has also been utilized to understand gambling problems [26]. When applied to PEAID, the model focuses on the interaction among individual characteristics (host), characteristics of the Internet and digital media itself (agent), and environmental factors, such as accessibility and social policy [27]. The public health model is consistent with the existing neuroscience-based model for problematic Internet use and allows us to expand the scope of intervention strategy. In the recently updated I-PACE model [28, 29], “rewards” in early stages and “cue-reactivity” in later stages can be viewed in terms of addictive feature of contents. In addition, “decision to behave in a specific way” in the early stages and “perception of external and internal triggers” in the later stages may be affected by sociocultural environments such as access to and provision of healthy pleasurable activities. Therefore, the public health model is useful for deriving social and technical intervention strategies beyond the existing neuroscience-based model.

Extensive evidence shows that each of the factors of the public health model influences PEAID. Intrapersonal and interpersonal factors have been the focus of considerable research pertaining to Internet addiction. Personal risk factors such as depression [30–32], anxiety [33, 34], aggression [35, 36], and impulsivity [37, 38] have been identified to increase the risk of Internet addiction. Interpersonal factors have also been found to be associated with Internet addiction. These include relationships with family members, family resilience, social bonds, and school functioning [39–41].

Agent factors related to the characteristics of the Internet and its influence on addictive behavior have received relatively less attention. However, agent factors inherent in alcohol or gambling have been examined in many neurobiological studies [42, 43]. Online games, similar to alcohol and gambling, stimulate the reward circuit among gaming addicts [35, 44]. The wide variety of Internet and digital media content leads users to engage in overuse. For instance, the sexual content, computer gaming, and gambling components of the Internet have been reported to be the most addictive aspects of the Internet [45–47].

Researchers have examined the seductive and gratifying properties of the Internet that attract users and found that certain properties elevate the risk of Internet addiction. These include self-presentation, relationship building, virtual community, and diversion [48–50]. Leung [50] pointed out that the pleasure of control and the perceived fluidity of identity are features of the Internet that are seductive to the Net Generation. A recent systematic review identified that game genre and structural characteristics contribute to the risk of problem gaming and gaming disorder [51]. Several researchers have suggested that the attraction of online games is an important risk factor for Internet addiction [52]. For instance, freedom, vividness, rewards, group identity, and avoidance of real-life problems have been identified as Internet game characteristics that increase the risk of addiction [53]. A study using a representative sample of Korean adolescents indicated that adolescents who perceived the Internet as a platform for socialization or building relationships were more likely to indulge in Internet and digital media use. The same study also identified that anonymity was an attractive property of the Internet related to addiction [27].

Social environmental factors associated with PEAID are of particular interest because of their relevance to policy and regulation development. Environmental factors such as Internet and Internet game access, sociocultural awareness, attitudes toward usage, exposure to advertisements, and lack of alternative leisure opportunities exert significant effects on Internet addiction. Te Wildt [54] contends that policies supporting faster and easier Internet access among young people can have a detrimental effect on Internet addiction. In a recent study, Korean adolescents’ ease of access to PC cafés was found to increase the risk of Internet addiction. Although PC cafés are relatively unique to Asian culture, the finding implies the influence of accessibility. In the area of alcohol research, several studies have confirmed the influence of advertisements on youths’ drinking attitudes and intentions [55, 56]. Applying this framework to Internet games, Chung et al. [27] found that adolescents who had more exposure to Internet game advertising and those who were more accepting of advertisements were at a higher risk of Internet addiction.

In summary, as the public health model posits, strategies directed at shifting intrapersonal, interpersonal, agent, and social environmental factors are needed to address PEAID. An awareness of the interplay among these factors will contribute to effective prevention, intervention, and policy development.

## Intervention Framework Based on the Public Health Model

The public health model focuses on addressing PEAID at both the individual and socio-environmental levels. At the same time, it recognizes the influence of the Internet or digital media as the agent. Intervention strategies based on the public health model should focus on reducing the risk factors in each domain.

First, considering the association between one’s individual vulnerability and Internet addiction, intervention strategies should take a mental health promotion approach to individuals that includes early identification and intervention as well as the development of appropriate prevention and treatment programs [12•].

Second, evidence has shown that certain features of the Internet and digital media, such as sexual content, gambling components, and games, increase the risk of problems and addiction. Policies need to target controlling exposure and accessibility to such content, especially for younger users. These may include warning signs, age regulations, and stronger monitoring systems.

Third, environmental factors (i.e., access to the Internet, attitude toward usage, permissive social culture, exposure to advertisements, aggressive marketing of the Internet and gaming industry) are usually related to the profit-seeking of related industries. Therefore, prevention and intervention efforts require public policies such as regulating excessive industry marketing, imposing stricter social responsibility measures on the industry, and developing systematic approaches for access restriction (i.e., shutdown, cooling off, and fatigue policies) [12•].

## Implementation of Comprehensive Intervention Strategy by Establishing the Global Information System on Digital Media and Health

**Fig. 1:**
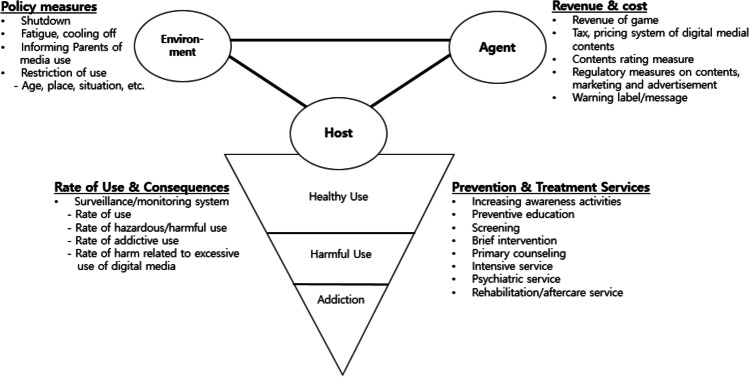
Comprehensive intervention strategy and Global Information System on Digital Media and Health

We have discussed how digital media overuse and addiction problems should be understood on a continuum similar to other types of addiction and how the problems of overuse and addiction occur as a result of the interaction among the three components of the public health model: the agent, host, and environment. Therefore, interventions based on the public health model should consider the following two aspects. One is to implement policy tools to reduce risks related to and increase protection against two components: the agent and environment. An environment-focused intervention may include restriction policies related to age, place, time, and circumstances. As mentioned above, some examples of these include a fatigue policy, PC café entry regulation, restriction of smartphone use while driving, and parent participation programs [19•]. Policies related to digital media content may include a content rating system, random item and obscene content regulation, information and warning provision (e.g., notification of digital media use time), and the use of technical devices (e.g., screen time applications) [57–59]. Another intervention strategy is to build an evidence-based intervention system that focuses on the individual (the host). Services for individuals require a systematic infrastructure in which services are provided according to problem type and severity based on the evaluation of digital media usage and patterns [60]. These include preventive measures such as education and increasing awareness at the early stage (i.e., before the development of excessive use), screening and early interventions for overuse, and intensive treatment provision for those already experiencing problems. Figure 1 depicts a conceptual model of policies and services to prevent PEAID based on the public health model. From the host perspective, the list provides indicators of digital media use and the degree of PEAID.

To establish an intervention strategy that encompasses these two aspects, objective data and indicators are necessary. More specifically, accurately assessing the amount and pattern of digital media use, the size of the risk group characterized by excessive use, the number of addicted persons experiencing functional impairment, and the context in which the harm occurs as well as the extent of the harm is important. Indeed, accurate understanding and monitoring of the problem are critical in the implementation of a public health strategy. Although the primary purpose of the international standard monitoring platform for digital media overuse, addiction problems, and intervention strategies is to understand the extent of the problem, it was also intended to list specific intervention strategies and provide guidelines for implementation. For instance, for tobacco and alcohol, the WHO has been striving to proliferate global evidence-based policies through an international surveillance and monitoring system. By instituting the Global Information System on Alcohol and Health (GISAH), the WHO has been monitoring and announcing public reports on alcohol consumption, related harm, an evidence-based policy and service system, and implementation [61]. Such a system not only accumulates information on alcohol consumption and related harm but also serves to recommend and disseminate a standardized list of evidence-based policies to be implemented to prevent related harm. Just as the public health model can be applied to digital media overuse and addiction, the same conceptual framework of the global information monitoring system can be applied to gaming and digital media. Table 1 shows a preliminary list of elements constituting the Global Information System on Digital Media and Health. The list can guide the identification of the current status of digital media use and related problems in each country and the monitoring of related policy measures that can facilitate implementation of evidence-based policies.

**Table 1:** Components of the Global Information System on Digital Media and Health

Area	Subcategory	Description
Surveillance & monitoring system	Rate of digital media use	According to age, sex, etc
	Frequency and amount of digital media use	According to age, sex, etc
	Type/genre of digital media use	Different content types
	Prevalence of problematic digital media use	High-risk and addictive use
	Rate of related consequences	Physical, mental, & behavioral
Sales & revenue	Total sales in digital media	Tax measure
	Tax and pricing	Price measure
Written policy	National policy regarding prevention of problems related to digital medial use	Develop national and local written measures
	Legislation regarding prevention of problems related to digital media use	Develop legislation
Policy according to related area	Accessibility control	
	Shutdown policy	Limit access by time
	Fatigue system	Limit efficiency of use
	Cooling off system	Automatic termination of use
	PC café entry	Restrict admission to PC cafés
	Addictive content control	
	Warning label	Provide information on risk
	Limitation of random items	Limit gambling potency
	Game rating system	Classify and limit content
	Game monetization	Limit monetization
	Provision of information	
	Inform time and money spent on digital media and/or game	Provide warnings about time and amount of use
	Inform parents of digital media use	Notify parents of use information
	Regulation on marketing	
	Restriction of advertisement	Limit place, time, subjects, etc
	Restriction of marketing	Limit place, time, subjects, etc
	Age or place limitation	
	Age limit on place, time, etc. of digital media use	Set limits on use according to age, time, place, etc
Prevention & treatment services	Education & primary prevention	
	Healthy use guidelines	Apply guidelines
	Awareness activity & campaign	Hold public activities to increase awareness
	School-based prevention education	
	Treatment	
	Screening & early intervention	Establish early screening & intervention system
	Counseling service for high-risk users	Provide readily accessible counseling services for high-risk users
	Treatment for high-risk users	Provide a clinic- & hospital-based treatment system for addicted users

As with collecting information on alcohol consumption and the prevalence of high-risk drinking and alcohol use disorder, collecting information on the extent and pattern of digital media use and the rate of pathological and addictive use to apply to policy implementation is possible. For instance, the shutdown policy and fatigue system parallel restricting the availability of alcohol. Limiting late-night access to PC cafés and regulating access to adult content for adolescents are equivalent to restricting alcohol sales to minors. Moreover, policies related to drinking and driving are similar to prohibiting the use of smartphones while driving [62]. Providing information and warnings about prolonged use corresponds to the mandatory warning labels on alcoholic beverages and restrictions on speculative and obscene content in digital media parallel regulating the alcohol content of alcoholic drinks [63]. In addition, regulations on the amount spent on gaming can be compared to the alcohol price policy [19•]. With the recent inclusion of GD in the ICD-11 and programs with proven effectiveness in providing healthcare services to individuals and groups [64••], the service system for alcohol use disorder can be directly applied to PEAID. The mandatory prevention education for the harmful use of alcohol may provide similar evidence for legalizing PEAID prevention education.

## Conclusion

Advances in digital technology and media have provided convenience and advantages in all areas of modern people’s daily lives. However, due to the readily accessible nature of the Internet, there is a risk of excessive and addictive use, which increases the risk of addiction as a disease and various mental and physical problems. These problems can deter development in children and adolescents in particular and are affecting many people. Therefore, interventions based on the public health perspective are necessary.

The public health model views addiction as the result of interactions among individuals, digital media, and the environment; therefore, the intervention should aim to reduce risk factors and increase protective factors in the three areas. In other words, in terms of the environment and media, accessibility restrictions and regulations according to the risk of content are representative approaches. This includes developing and providing evidence-based services according to each individual’s problem severity and type.

This calls for an integrative, comprehensive, and continuous intervention strategy, and to achieve such a framework, we need to establish an information system to monitor the magnitude and patterns of related problems. Such a monitoring and surveillance system will be able to provide a list of evidence-based policies based on the public health perspective. We propose a structure similar to the GISAH enacted by the WHO. To reduce the global problem related to the excessive use of the Internet and digital media and to achieve a sustainable digital society, we need to institute an advanced international information, policy, and monitoring system.
